# Pathogenesis and Manifestations of Zika Virus-Associated Ocular Diseases

**DOI:** 10.3390/tropicalmed7060106

**Published:** 2022-06-15

**Authors:** Bisant A. Labib, DeGaulle I. Chigbu

**Affiliations:** Pennsylvania College of Optometry, Salus University, Elkins Park, PA 19027, USA; blabib@salus.edu

**Keywords:** Zika virus, virology, infectious disease, conjunctivitis, anterior uveitis, chorioretinitis, glaucoma

## Abstract

Zika virus (ZIKV) is mosquito-borne flavivirus that caused a significant public health concern in French Polynesia and South America. The two major complications that gained the most media attention during the ZIKV outbreak were Guillain–Barré syndrome (GBS) and microcephaly in newborn infants. The two modes of ZIKV transmission are the vector-borne and non-vector borne modes of transmission. Aedes aegypti and Aedes albopictus are the most important vectors of ZIKV. ZIKV binds to surface receptors on permissive cells that support infection and replication, such as neural progenitor cells, dendritic cells, dermal fibroblasts, retinal pigment epithelial cells, endothelial cells, macrophages, epidermal keratinocytes, and trophoblasts to cause infection. The innate immune response to ZIKV infection is mediated by interferons and natural killer cells, whereas the adaptive immune response is mediated by CD8^+^T cells, Th1 cells, and neutralizing antibodies. The non-structural proteins of ZIKV, such as non-structural protein 5, are involved in the evasion of the host’s immune defense mechanisms. Ocular manifestations of ZIKV arise from the virus’ ability to cross both the blood–brain barrier and blood-retinal barrier, as well as the blood-aqueous barrier. Most notably, this results in the development of GBS, a rare neurological complication in acute ZIKV infection. This can yield ocular symptoms and signs. Additionally, infants to whom ZIKV is transmitted congenitally develop congenital Zika syndrome (CZS). The ocular manifestations are widely variable, and include nonpurulent conjunctivitis, anterior uveitis, keratitis, trabeculitis, congenital glaucoma, microphthalmia, hypoplastic optic disc, and optic nerve pallor. There are currently no FDA approved therapeutic agents for treating ZIKV infections and, as such, a meticulous ocular examination is an important aspect of the diagnosis. This review utilized several published articles regarding the ocular findings of ZIKV, antiviral immune responses to ZIKV infection, and the pathogenesis of ocular manifestations in individuals with ZIKV infection. This review summarizes the current knowledge on the viral immunology of ZIKV, interactions between ZIKV and the host’s immune defense mechanism, pathological mechanisms, as well as anterior and posterior segment findings associated with ZIKV infection.

## 1. Introduction

**Figure 1:**
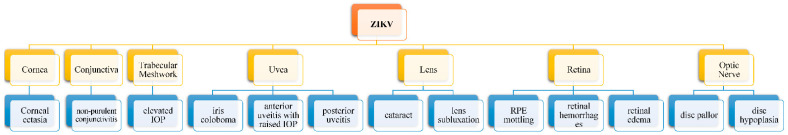
Ocular manifestations of Zika virus infection.

Zika virus (ZIKV) is a mosquito-borne flavivirus of global medical importance that belongs to the Flaviviridae family. Other flaviviruses include dengue virus, hepatitis C virus, yellow fever virus, Japanese encephalitis virus, and West Nile virus. ZIKV acquired global health significance when it caused significant outbreaks in Yap Island, French Polynesia, and South America [1]. ZIKV was identified and isolated from a rhesus monkey in Zika forest in Uganda in 1947 [2,3]. There have been a few isolated cases of Zika virus infections prior to the ZIKV-associated outbreak that occurred in the Federated States of Micronesia, which was followed by an outbreak in French Polynesia in 2013 and South America in 2015 [4]. Many cases of Guillain–Barré syndrome were observed during the ZIKV outbreak in French Polynesia, whereas microcephaly in newborn infants was associated with the ZIKV outbreak in South America [1]. Ocular findings associated with congenital Zika syndrome include iris coloboma, microphthalmia, optic disc pallor, chorioretinal atrophy, lens subluxation, cataract, glaucoma, strabismus, retinal pigment epithelial mottling, maculopathy, abnormal retinal vessels, and pupillary abnormalities. However, pruritic maculopapular rash, transient low-grade fever, arthralgia, anterior uveitis, chorioretinitis, trabeculitis, and nonpurulent conjunctivitis are commonly observed clinical expressions of acute ZIKV infection (Figure 1) [1,5,6]. There are two modes of transmission of ZIKV. The vector-borne mode of transmission is the most common form of ZIKV transmission through the bite of infected Aedes aegypti and Aedes albopictus mosquitoes [1,7]. The non-vector mode of transmission involves the transmission of ZIKV from human-to-human, such as vertical transmission between mother and fetus, sexual intercourse, and breastfeeding [1,8,9]. Other non-vector modes of ZIKV transmission include ocular transmission and blood transfusion. Ocular transmission can potentially occur when there is contact with conjunctival fluid and tears [10]. Tan and coworker [11] suggested that contact with ocular discharge, such as tears of a patient with ZIKV infection, can serve as a non-vector mode of transmitting ZIKV. The persistence of ZIKV RNA in tears 30 days post-illness has been documented. Thus, there is a potential risk of ocular transmission of ZIKV through contact with ZIKV infected tears during the high viremic phase of ZIKV infection [11,12]. Because of the presence of ZIKV in vaginal secretions, there is also a possibility of a female-to-male transmission of ZIKV and maternal transmission during vaginal delivery [7]. This review will summarize the current knowledge on the viral immunology of ZIKV, interactions between ZIKV and the host’s immune defense mechanisms, pathological mechanism, anterior and posterior segment ocular findings, as well as neuro-ophthalmic disorders associated with ZIKV infection.

## 2. Virology

Zika virus is a positive-sense enveloped RNA virus with a genome of ~11 kilobases, open frame enclosed in an icosahedral nucleocapsid [13,14]. The envelope protein of ZIKV binds to surface receptors on the target host cell to initiate the process of attachment, fusion, and entry. Clathrin-mediated endocytosis is utilized by ZIKV to gain access to the endosome of the host cell, where changes in the acidic pH of the endosome is associated with fusion between the viral envelope and the endosomal membrane that facilitates the release of ZIKV into the cytoplasm. This is followed by an uncoating process to release the viral RNA genome into the host cell cytoplasm [1,15,16]. The uncoating of the virus occurs in the cytoplasm in order to release the viral RNA genome for transcription and replication [1,15,16,17]. The positive-sense genome serves as viral messenger RNA that undergoes translation to yield a single polyprotein that in turn undergoes co- and post-translational cleavage by host cellular and viral proteases to generate three structural proteins and seven non-structural (NS) proteins [18,19,20]. Capsid protein is a structural protein that serves as the nucleocapsid, whereas the pre-membrane protein is complexed to the envelope protein. The pre-membrane protein prevents the premature fusion of ZIKV to the membrane of the host cell [14]. The envelope protein (E protein) is a highly conserved protein in all members of the Flaviviruses. The three domains of the E protein are envelope domain I (EDI), EDII, and EDIII. EDIII is responsible for mediating the attachment of the virus to receptors on permissive host cells. Antibodies generated against EDIII are the most potent neutralizing antibodies [21]. While the ZIKV envelope protein plays a role in viral assembly, it also acts as a viral attachment protein that facilitates attachment, entry, and fusion of the virus [14]. The nonstructural proteins are NS1, NS2a, NS2b, NS3, NS4a, NS4b, and NS5. The N-terminal of NS3 along with NS2B form a NS2B–NS3 protease complex that cleaves the polyprotein. The C-terminal of NS3 protein has helicase and nucleoside–triphosphate (NTPase) activities, and it participates in viral RNA synthesis [22,23]. NS5 is a conserved, non-structural protein that contains the RNA-dependent RNA polymerase required for viral replication. It also houses the methyltransferase domain, which is required for evading the immune response [24]. NS5 binds to STAT2 to induce the proteasomal degradation of STAT2, which results in STAT1-SATA1 homodimerization, a protein complexes that acts on genes that promote the production of type II IFN [10,25]. NS5 also blocks type I and III IFN-mediated antiviral response [26]. NS2A, NS4A, and NS4B play a role in viral replication and assembly [24]. NS4A also aids in polyprotein processing and immune evasion [27].

## 3. Host–Virus Interaction

Astrocytes, dendritic cells, dermal fibroblasts, endothelial cells, monocytes, macrophages, microglia, neural stem cells, mesenchymal stem cells, neural crest cells, neurons, epiderma keratinocytes, and trophoblasts are cells that support the infection and replication of ZIKV, as well as the subsequent dissemination of the virus to other permissive cells and tissues [28,29,30]. When an Aedes mosquito takes a blood meal from an infected individual it becomes infected and capable of transmitting ZIKV to uninfected humans during future blood meals. The virus interacts with TYRO3, AXL, and TIM-1 expressed on epidermal keratinocytes and dermal fibroblasts to gain access to, infect, and subsequently replicate within these cells [16,31].

### 3.1. Innate Immune Response to ZIKV Infection

**Figure 2:**
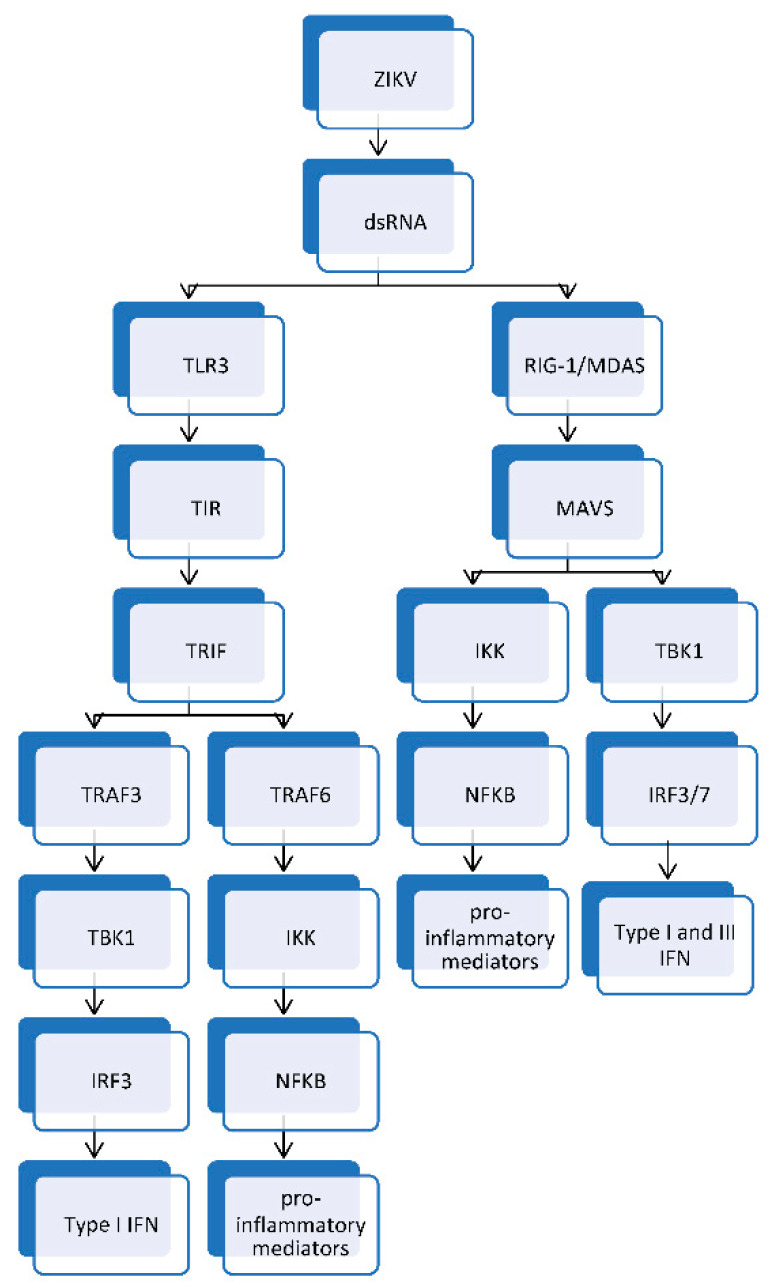
TLR3-mediated innate immune response yields type I IFN and proinflammatory mediators. The RIG-I/MDA5 signaling pathway generates type I IFN, type III IFN, and proinflammatory mediators. TLR3: Toll-like receptor 3; RIG-I: retinoic acid-inducible gene I; MDA5: melanoma differentiation-associated gene 5; MAVS: mitochondria antiviral signaling; TIR: toll/interleukin-1 receptor; TRIF: TIR-domain-containing adapter-inducing interferon-β; TRAF3: tumor necrosis factor receptor associated factor 3; TRAF6: tumor necrosis factor receptor associated factor 6; TBK1: TANK-binding kinase 1; IKK: Inhibitory kappaB kinase; IRF3/7: Interferon regulatory factor 3/7; IRF3: Interferon regulatory factor 3; NF-κB: nuclear factor-kappaB; Type I IFN: Type I interferon; Type III IFN: type III interferon.

ZIKV engages DC-SIGN expressed on macrophages and dendritic cells to facilitate the infection of these activators and sentinel cells of the immune system [16,31]. The presence of ZIKV results in pattern recognition receptors of the innate immune system to engage pattern associated molecular patterns of ZIKV. The activation of the complement system by flaviviruses, including ZIKV, through the alternative, lectin, and classical pathways culminates in the generation of C3a, C3b, C5a, and the membrane attack complex (MAC). C3a and C5a mediate the recruitment of immune cells to the site of complement activation. This is accomplished by C3a and C5a engaging their receptors on vascular endothelium to trigger the upregulation of ICAM-1 on vascular endothelium to enhance vasopermeability, which facilitates the influx of immune cells to the site of complement activation. The MAC lyses enveloped viruses, such as members of the flaviviruses [32,33]. Nonstructural proteins, such as NS1, play a role in the replication of ZIKV within the host; however, it can play a major role in mediating the viral pathogenesis and evasion of the innate immune response [24]. NS1 can bind and recruit complement factor H (CFH), which, in turn, results in the viral-mediated dissociation of C3 convertase of the alternate pathway of complement. This culminates in a dysfunction of the alternative pathway of complement with little or no formation of complement opsonins, such as C3b, as well as a reduction in the generation of chemoattractants of the complement system, such as C3a and C5a [34]. Furthermore, ZIKV can utilize the NS1 to bind to regulators of the complement system, such as vitronectin, to escape complement mediated lysis by preventing the formation of the MAC on the viral envelope [35]. Additionally, ZIKV NS1 can decrease the polymerization of C9 in order to evade complement-mediated lysis of ZIKV [24,35]. Malekshahi and coworkers [35] demonstrated that the ZIKV envelope protein can bind to and incorporate vitronectin into its envelope to prevent complement-mediated lysis of the virus. ZIKV E protein binds to the terminal components of the complement system. It also reduces the polymerization of complement protein 9 (C9) in order to prevent the formation of the membrane attack complex. Thus, it is able to inhibit complement-mediated lysis [24,35]. In addition to the role of complement in innate immune response against ZIKV, innate immune response is also mediated by type 1 interferon (IFN), type 2 IFN, type 3 IFN, macrophages, and natural killer (NK) cells. Dendritic cells, macrophages, keratinocytes, and fibroblasts have pattern recognition receptors, such as Toll-like receptor 3 (TLR3), retinoic acid-inducible gene I (RIG-I), and melanoma differentiation-associated gene 5 (MDA5). The RIG-I-like receptor (RLR) is a nucleic acid receptor present in the cytosol of host cells and acts as an innate immune sensor of pattern associated molecular patterns (PAMPs) expressed by ZIKV. It is of note that following the entry of ZIKV into the cytosol of the permissive target cell, it undergoes uncoating to expose and release the viral RNA genome for transcription and replication. The viral ssRNA genome undergoing transcription and replication to yield dsRNA, a replication intermediate that binds to RIG-I and MDA5 [36,37,38]. The interaction between dsRNA and RIG-I/MDA5 leads to conformational changes that initiate downstream signaling cascades, which results in the two N-terminal caspase-recruitment domains (CARD) of RIG-I and MDA5 binding to the CARD domain of a downstream adaptor protein mitochondria antiviral signaling protein (MAVS) to mediate the recruitment and activation of MAVS. The activated MAVS recruits and activates downstream signaling protein kinase TANK-binding kinase 1 (TBK1) and inhibitory kappaB kinase (IKK). TBK1 and IKK phosphorylate interferon regulatory factor 3/7 (IRF3/7) and nuclear factor-kappaB (NF-κB), respectively. Phosphorylated IRF3/7 dissociates, dimerizes, and translocates to the nucleus to work along with NF-κB to produce type I and III IFN and other cytokines (Figure 2) [19,37,38,39].

Toll-like receptors (TLR) serve as pattern recognition receptors that reside in the endosome of host cells. TLR3 recognizes dsRNA, a replication intermediate, and this interaction is associated with a downstream signaling cascade, in which the toll/interleukin-1 receptor (TIR) domain of TLR3 binds to the (TIR) domain of TIR-domain-containing adapter-inducing interferon-β (TRIF). This interaction between TLR3 and TRIF induces the recruitment and activation of TRIF. This is followed by the binding of the activated TRIF to tumor necrosis factor receptor associated factor 3 (TRAF3) and TRAF6. TRAF3 engages and recruits TBK1, resulting in the activation of IRF3. TRAF6 engages and recruits IKK and this results in the activation of NFκB. IRF3 and NFκB translocate to the nucleus where they stimulate genes required for coding interferons (type I IFN) and other cytokines respectively (Figure 2) [20,38,39,40,41].

**Figure 3:**
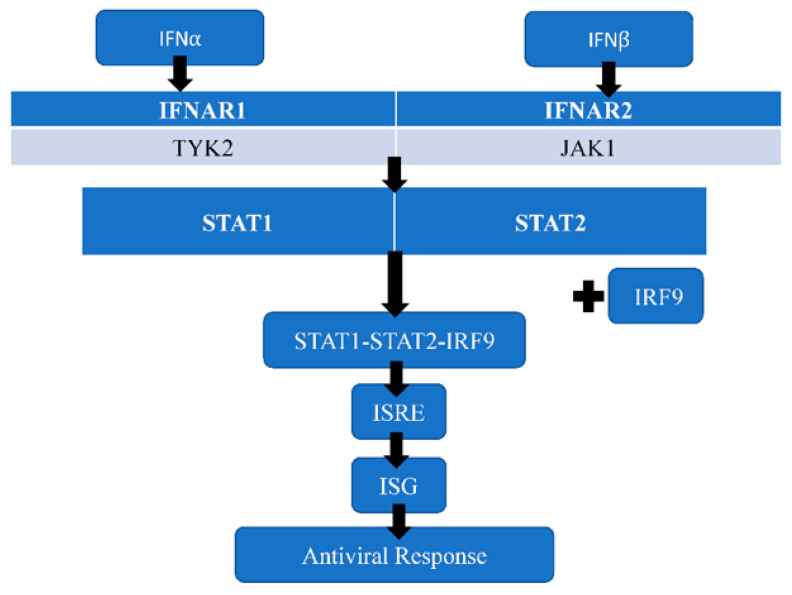
IFNα/β generates the antiviral response to control ZIKV infection. IFNα: Interferon alpha; IFNβ: Interferon beta; IFNAR1: interferon alpha receptor 1; IFNAR2: interferon alpha receptor 2; TYK2: Tyrosine kinase 2; JAK1: Janus activated kinase 1; STAT1: signal transducer and activator of transcription 1; STAT2: signal transducer and activator of transcription 2; IRF9: interferon regulatory factor 9; ISG: Interferon-stimulated genes.

Interferons are one of the major host restriction factors that inhibit the replication of ZIKV. Type I interferons (IFNI or IFNα/β) produced by ZIKV-infected cells induce an antiviral microenvironment by interacting with IFNα/β receptors (type I IFN receptor), a type 2 cytokine receptor. Type I IFN receptors consist of interferon alpha receptor1 (IFNAR1) and IFNAR2 subunits that project into the cell to form a cytoplasmic domain associated with Janus activated kinases, such as tyrosine kinase 2 (TYK2) and Janus activated kinase 1 (JAK1) [42,43]. The binding of type I IFN to its cognate receptor results in a signal cascade that activates JAK1 and TYK2. The activated Janus activated kinases phosphorylate tyrosine residues on the cytoplasmic domain to provide a docking site for signal transducer and activator of transcription 1 (STAT1) and STAT2 proteins. STAT1 and STAT2 proteins are recruited by the activated JAKs, and subsequently they undergo phosphorylation and dimerization. The phosphorylated STAT1 and STAT2 proteins dissociate from JAKs and bind to interferon regulatory factor 9 (IRF9) to form the STAT1–STAT2–IFR9 complex, a trimolecular complex called interferon-stimulated gene factor 3 (ISGF3). ISGF3 translocates into the nucleus of the host cell, where it binds to IFN-stimulated response element (ISRE) on DNA to trigger the transcription of interferon-stimulated genes (ISG) that induce an antiviral response to control the ZIKV infection (Figure 3) [42,43]. Thus, the innate immune response against ZIKV results in increased expression of RIG-I, MDA5, and TLR3 [10]. TLR3 engages dsRNA intermediates to induce the activation of IRF3 and NFκB, whereas RIG-I/MDA5 engages dsRNA intermediates to induce the activation of IRF3/7 and NFκB [25]. This interaction yields type I IFN, IL-6, IL-12, tumor necrosis factor alpha (TNFα), type III IFN, ISG, CXCL10, and CCL5 [10,25].

ZIKV, like most members of the flaviviruses, evades host immune responses by interfering with molecules that mediate innate immunity. NS1, NS4, and NS4B inhibit the signaling pathway essential for the generation of IRF3 and IRF7. NS4 inhibit the activity of MAVS, whereas NS1 and NS4B inhibit the activity of TBK1 [24]. However, it has been reported that NS5 inhibits the generation of type 1 IFN by mediating the degradation of STAT2 [4]. NS1, NS4B, and NS2B are involved in the process of mediating the evasion of the immune system, which is accomplished by blocking the activation of type I IFN and expression of ISG [19].

Type 1 IFN induces an antiviral microenvironment to limit the spread of the virus, as well as activate NK cells that mediate cytolytic and noncytolytic antiviral immune responses. Perforin and granzyme secreted by activated NK cells induce the cytolysis of ZIKV infected cells. NK cells secrete type II IFN (IFNγ) that inhibit viral replication and activate macrophages. Furthermore, IFNγ and TNFα secreted by activated NK cells facilitate the maturation and antigen presentation capability of dendritic cells [44].

### 3.2. T Cell-Mediated Immune Response to ZIKV Infection

Dendritic cells, loaded with viral peptides derived from ZIKV complexed to major histocompatibility complexes, migrate to the regional lymph node to activate naïve T cells. Activated T cells undergo proliferation and differentiation into effector T cells, such as effector CD4^+^T cells and effector CD8^+^T cells. IL-12 (secreted by dendritic cells and macrophages) and type II IFN (secreted by NK cells) induce activated CD4^+^T cells to differentiate into Th1 cells that secrete IL-2, IFNγ, and TNFα [45,46]. Hassert and coworkers used a mouse model of ZIKV infection to demonstrate that ZIKV epitope-specific CD4^+^T cells induced protective cell-mediated immune responses with Th1 cells secreting type 2 IFN and TNFα, being the predominant subset of effector CD4^+^T cells [47]. In studying the immunological features of ten women with acute ZIKV infection, Tonnerree and coworkers revealed that CD4^+^T cells were shown to target capsid protein, pre-M protein, Envelop protein, NS1, NS3, and NS5 proteins. In these particular patients, CD4^+^T cells generated both effector-memory CD4^+^T cells and central-memory CD4^+^T cells. Memory CD4^+^T cells and CD8^+^T cells produced TNFα and IFNγ [48]. CD8^+^T cells mediate cell-mediated cytotoxicity through perforin and granzyme. These granules induce the cytolysis of ZIKV-infected cells. Additionally, IFNγ and TNFα secreted by effector CD8^+^T cells mediate noncytolytic antiviral immune responses against ZIKV [49]. Ngono and coworkers, using mouse models, were able to demonstrate that CD8^+^T cells target all ZIKV proteins except NS1 and NS2B [50]. Tonnerree and coworkers also revealed that CD8^+^T cells targeted nonstructural proteins of ZIKV [48].

### 3.3. Antibody-Mediated Immune Response to ZIKV Infection

Zika virus induces the generation of a humoral immune response with ZIKV-specific B cells, producing ZIKV-specific plasma cells that secrete IgG and IgA. IgM is produced during the primary infection with the subsequent generation of high affinity antibodies with potent opsonizing capabilities against the structural proteins of ZIKV [51,52]. Antibodies generated against EDIII during ZIKV infections are potently neutralizing antibodies that can provide protective immunity [21]. Antibodies developed against non-structural proteins, pre-membrane, and EDI/II of ZIKV have little or no neutralizing capabilities and are usually cross-reactive antibodies. Thus, EDI/II-reactive antibodies generated against other members of the Flaviviruses demonstrate little/no neutralizing effect against ZIKV [53,54,55]. It is noteworthy that the generation of humoral immunity against ZIKV EDIII is associated with the generation of ZIKV EDIII memory B cells and long-lived plasma cells that continuously secrete ZIKV EDIII-specific antibodies [21].

## 4. Ocular Manifestations

The ocular manifestations of ZIKV infection differ depending on acute infections versus congenital forms. In either case, ocular findings arise from the virus’s ability to cross both the blood–brain barrier and blood-retinal barrier, as well as the blood-aqueous barrier [56]. In acute ZIKV infection, there is no definitive outline of symptoms. They vary from anterior segment findings of conjunctivitis and anterior uveitis to posterior segment findings of maculopathy with outer retinal layer and retinal pigment epithelium (RPE) disruption (Table 1) [8,57,58,59,60,61]. The most common ocular manifestation in acute ZIKV infection is a nonpurulent conjunctivitis, arising in up to 63% of patients [62]. The data differs in pregnant women with ZIKV who did not show evidence of associated conjunctivitis [63]. The other potential anterior segment manifestation of acute ZIKV infection is anterior uveitis with raised intraocular pressure. In one study, a 39-year-old man in Brazil, diagnosed with ZIKV based on clinical findings alone, developed bilateral iridocyclitis with elevated intraocular pressures [64]. Similarly, a middle-aged man with a confirmed diagnosis of ZIKV through aqueous humor analysis had a resultant bilateral nongranulomatous anterior uveitis [57]. A posterior segment finding in one case report in 2019 describes a unilateral multifocal choroiditis, presumably related to acute ZIKV infection [65].

A minority of those who are infected with ZIKV often have mild, non-specific symptoms, such as a low-grade fever, myalgia, or headache [2,68]. In rare cases, patients develop Guillain–Barré syndrome (GBS), a condition that causes damage to the peripheral nervous system [2]. The immunopathological mechanism of ZIKV-associated GBS is attributed to an autoimmune process involving molecular mimicry, in which cross reaction occurs due to the similarities between immunogenic epitopes of ZIKV polyprotein and host neuronal membrane gangliosides [69,70]. In this subset of patients, there was one case of post-infectious papilledema reported. The incidence of acute ZIKV infection causing GBS has increased over time. There was a large outbreak in French Polynesia involving 42 patients who all had GBS and acute ZIKV infection [71]. Furthermore, those who develop Guillain–Barré and have had previous exposure to dengue virus may develop Miller Fisher Syndrome, a rare variant of GBS in both dengue virus and ZIKV, which causes ophthalmoplegia [5,66]. Another case report describes a 22-year-old female with ZIKV infection and subsequent Guillain–Barré syndrome who also developed ocular flutter. This rare ocular dyskinesia is potentially a response to ZIKV infection [67] and occurs otherwise in paraneoplastic disorders, encephalitis, serotonin syndrome, and other infections, such as hepatitis C viral infections [72]. Ocular flutter has also been associated with enteroviral infections [73] and dengue viral infections [74].

On the other hand, congenital Zika syndrome (CZS) manifests with a wide variety of well documented ocular manifestations. Ocular manifestations occur in up to 50% of infants who have ZIKV-related microcephaly [63,75]. The CZS associated ocular findings affect both anterior and posterior segment structures (Table 2). Anterior segment findings include iris coloboma, lens subluxation, cataract, glaucoma, microphthalmia, and intraocular calcifications. Neuro-ophthalmic complications may also arise, related to oculomotor and abducens muscle paresis, which manifest clinically as convergent strabismus and loss of pupillary response [63,76,77,78]. Posterior segment abnormalities, if present, affect the retina and choroid, often mimicking toxoplasmosis, appearing as macular pigment mottling and chorioretinal atrophy [5,79].

The first ophthalmic findings in CSZ were documented in 2016 by Ventura et al. [79], who described three infants with unilateral loss of foveal reflex as well as macular pigment mottling. They later described one child with horizontal nystagmus, four with exophoria, and two with esophoria. Most eyes (85%) had both macular and optic nerve abnormalities. In addition to the aforementioned macular findings, distinct areas of chorioretinal atrophy were also observed. Optic nerve abnormalities included hypoplastic disc, nerve pallor, and an increased cup-to-disc ratio [79]. These findings were consistently documented in studies thereafter [89,90,91]. De Paula Freitas observed additional findings, including lens subluxation and iris coloboma. Posterior segment findings in this study were consistent with previous data and confirmed the presence of macular, paramacular, or nasal chorioretinal atrophy as a finding of CZS [63].

Additional studies in different regions in Brazil confirmed these findings later on in 2017 by de Oliveira and Zin [86,92]. However, Zin et al. reported the presence of retinal hemorrhage, which was not previously mentioned. They also concluded that ocular findings may only be the initial finding of CZS, emphasizing the importance of early examination [86].

An additional study on the posterior segment manifestations of CZS described similar presentations, as well as the presence of vascular tortuosity, early termination of retinal vasculature, washed out peripheral retinal peripheries with a hypoluscent spot, scattered paramacular hemorrhages, and peripheral pigmentary changes with clustered hypopigmented lesions. In a little less than half of cases, optic nerve abnormalities were also present. These included either disc hypoplasia, pallor, or large cupping [85]. Though anterior segment findings in CZS were largely absent, one study did report one case of bilateral iris colobomas and unilateral lens subluxation [61,63].

Yepez et al. documented a case series from Venezuela with many of the aforementioned ocular findings. However, in this case series, 12% of infants also had congenital glaucoma [80]. The presence of congenital glaucoma related to ZIKV was first observed in a case documented in 2017 by de Paula Freitas, in addition to retinal findings [93]. In all patients with congenital glaucoma described by Yepez et al., epiphora, photophobia, blepharospasm, increased IOP, buphthalmos, and corneal enlargement and clouding were present to make the diagnosis. A few patients also presented with Haab’s striae and enlarged cup-to-disc ratios. Infants in this study required invasive surgical intervention to treat the glaucoma [80].

Regardless of retinal or macular findings, all infants had decreased visual acuity. In a study of 32 infants with CZS, Teller acuity was used to measure visual function, and found that all had reduced vision. Retinal findings, however, were present in less than half. This is presumably due to the CNS involvement associated with CZS. Decreased visual acuity was confirmed in a later study but improvement was observed by prescribing full visual correction in these cases [90].

A study comparing optical coherence tomography (OCT) findings in CZS found that all infants exhibited the discontinuation of the ellipsoid zone and hyper-reflectivity underlying the retinal pigment epithelium. The majority of infants in the study also showed retinal and choroidal thinning on OCT, with some also manifesting with chorioretinal atrophy resembling coloboma [87].

While fundus abnormalities are the most common manifestation of CZS, there are some virus-related anterior segment signs as well, such as microphthalmia [81,82]. One case report describes a child with a history of presumed ZIKV infection at 11 weeks’ gestational age with corneal ectasia, which may be an additional anterior segment finding of CZS. This corneal involvement can be explained by studies wherein Zika virus RNA has been found in the cornea of mice, as well as neurosensory retina and optic nerve [83]. Microphthalmia, iris coloboma, and cataracts can all occur in CZS. One study suggests that the presence of iris coloboma is potentially a marker for infection that occurred prior to the seventh week of gestation, where there is closure of the optic fissure. Furthermore, in a small subset of infants with iris coloboma, the location was either superior or temporal, which is atypical [84].

Histopathological studies on eyes of post-mortem fetuses with CZS revealed anatomical anterior segment abnormalities, including pupillary membrane and anterior chamber angles that are not fully formed. Retinal and choroidal abnormalities included thin photoreceptor layer, RPE thinning and pigment loss, choroidal perivascular inflammatory infiltrate, and choroidal thinning [88].

Interestingly, the mothers of children with CZS do not develop ocular abnormalities. However, these infants primarily present with the retinal findings of unilateral pigment mottling or chorioretinal atrophy [85]. Ocular involvement was more likely to occur in babies of mothers who were infected with ZIKV within the first trimester of pregnancy [63]. It is difficult to ascertain which infants with ZIKV-infected mothers will develop CZS, due to the lack of ophthalmic manifestations and/or symptoms in affected mothers during pregnancy. One study suggested that infants born with microcephaly had a higher risk of developing ophthalmic manifestations [94].

## 5. Pathological Mechanisms

Viral cellular tropism is determined by the ability of ZIKV to bind to the host cell surface receptors [95]. Dendritic cell-specific intercellular adhesion molecule-3 grabbing nonintegrin (DC-SIGN) or CD209 are expressed on dendritic cells and macrophages [96,97]. TIM-1 (T-cell immunoglobulin and mucin domain 1) is an attachment molecule for ZIKV [28,29], whereas TAM (TYRO3, AXL and MER) is a receptor tyrosine kinase that serves as a cellular receptor for the ZIKV family of receptors [28,29,98]. AXL and TYRO3 are expressed on retinal pericytes, retinal microvascular endothelial cells [24], and neural progenitor cells (NPCs) [99]. Furthermore, TYRO3, AXL, and TIM-1 are expressed on human placenta cells, endothelial cells, trophoblast cells, fibroblasts, trophoblast progenitor cells, macrophages [27,29], epidermal keratinocytes, and dermal fibroblasts [96]. Additionally, AXL and TYRO3 facilitate the ZIKV infection of astrocytes and microglia cells [95] whereas AXL facilitates the infection of neural stem cells [99], neural crest cells [100], and mesenchymal stem cells [101]. Pericytes, RPE cells, and microvascular endothelial cells of the retina are permissive cells that support the infection and replication of ZIKV [102]. ZIKV infects NPCs, leading to the apoptosis of NPCs, while also inhibiting the differentiation of NPCs. This culminates in developmental delay due to the depletion of the NPCs, as observed in the prenatal brain [99].

Viral persistence has a role to play in the pathogenesis of ZIKV infection. It has been suggested that ocular immune privileged sites are prone to ZIKV-induced changes because of viral persistence due to the high viral load. The cells of these blood–tissue barriers could serve as a reservoir for ZIKV, they also serve as a medium for facilitating the spread of the virus [103]. Although the immune system of the host is responsible for protecting the host from viral diseases, an overactive immune response can be a contributory factor to viral pathogenesis [104]. ZIKV can gain access to the eye through retrograde transport of ZIKV through the optic tract and optic nerve into the eye, as well as through hematogenous spread of ZIKV across the blood–ocular barriers [105].

### 5.1. Blood–Tissue Barriers

Blood–tissue barriers, such as the blood–brain barrier (BBB), maternal–fetal interface, blood–retinal barrier (BRB), and blood–aqueous barrier (BAB), have cells that are vulnerable to infection by ZIKV. Endothelial cells of the BBB are vulnerable to ZIKV infection and serve as a reservoir that facilitates dissemination of ZIKV via the blood–brain barrier. This allows ZIKV access to cell types, such as neural stem cells (NSCs), neurons, astrocytes, and microglia [106]. The endothelial cells of the BBB and BRB cells can secrete matrix metalloproteinase-2 (MMP-2) and MMP-9 that are responsible for disrupting the impermeability function of BBB and BRB to facilitate access of infiltrating effector immune cells into the brain and retina, respectively [107]. ZIKV can cause a breakdown of the RPE impermeability function by impairing the intercellular junctions that link up the individual RPE cells that constitute the external BRB [105]. MMP-2 and MMP-9 secreted by ZIKV-infected RPE cells can degrade the intercellular junctional proteins, which culminates in impairing the RPE impermeability function [105,108]. The choriocapillaries supply blood to the retina. The highly fenestrated choriocapillaries have endothelial cells that are prone to infection by ZIKV, and the infection of endothelial cells of the choriocapillaries can facilitate the access of ZIKV to the highly permissive RPE. Infection of RPE cells induce their activation and promote inflammation that breaks down the blood–retinal barrier [109].

### 5.2. ZIKV-Infected Myeloid Cells

The Hofbauer cells (HBCs) are fetal macrophages that increase in number during ZIKV infection and are susceptible to direct infection by ZIKV [10]. ZIKV-infected HBCs can serve as a reservoir for ZIKV and act as a Trojan horse that facilitates the dissemination of ZIKV into the fetal blood [7]. Furthermore, ZIKV-infected monocytes in the blood stream can pass through the blood–placenta barrier to gain access to the fetus, where they invade and infect the fetal neural tissue due to the neurotropism of ZIKV. Additionally, ZIKV-infected monocytes have the ability to transmigrate the retinal microvascular endothelium to facilitate the dissemination of ZIKV to cells in the neuroretina and other immune-privileged sites of the eye. ZIKV infected monocytes become activated to secrete cytokines and chemokines that promote inflammation in these immune-privileged sites. Thus, ZIKV-infected monocytes act as Trojan horses that play a dual role in ZIKV pathogenesis via the dissemination of the virus and initiating inflammation in immune-privileged tissues [110,111].

### 5.3. Microcephaly

ZIKV infection during the early stages of CNS development is associated with a greater risk of developing microcephaly. The risk is highest in the first trimester of pregnancy [112]. Microcephaly, a neurodevelopmental disorder, is characterized by a significant reduction in the size of the brain. It is caused by the impaired proliferation of NPCs and death of NPCs [26]. ZIKV-infected NPCs undergo apoptosis through the activation of p53, which results in a reduction in the number of NPCs. The p53-mediated apoptosis is usually associated with the TLR3-mediated activation of the innate immune response [113]. Furthermore, in ZIKV-infected cells, mitochondrial fragmentation occurs prior to the apoptosis of ZIKV-infected cells. It has been demonstrated that during ZIKV infection the mitofusin 2 (MFN2) protein required to mediate mitochondrial fusion is reduced, and as such, mitochondria fragmentation is involved in the mitochondria apoptotic pathway observed in ZIKV infection [113,114]. In the ZIKV-infected fetal brain, there is a reduction in the proliferation of NPCs with a consequential reduction in the number of NPCs [115]. The remainder of the NPCs will undergo premature differentiation into mature neurons [113]. Thus, impaired neurogenesis secondary to depletion of NPCs and premature differentiation of ZIKV-infected NPCs causes a reduction in the brain size, which manifests as ZIKV-related microcephaly (Table 3) [115,116,117,118]. Immune responses mounted against ZIKV during pregnancy are associated with congenital malformations due to the immune-mediated inflammation at the maternal–fetal interface that compromises the barrier function of this interface. This allows effector immune cells, including IFNγ and TNFα secreting effector T cells, to gain access to the fetus, where these cells promote inflammatory responses that destroy NPCs required for the normal neural development of the fetus [25]. ZIKV infection of blood–brain barrier cells, such as endothelial cells, pericytes, and astrocytes, can cause an upregulation of ICAM-1 that promotes the efficient docking of effector immune cells to the blood–brain barrier, thereby contributing to the influx of effector immune cells [119]. ZIKV-infected blood–brain barrier cells also upregulate the expression of IL-6, CCL5, and CXCL10, which mediates the chemotaxis of effector immune cells to the blood–brain barrier. The recruited immune effector cells engage the ICAM-1 expressed on the infected blood–brain barrier cells and transmigrate through the blood–brain barrier to gain access to central nervous system to cause neuroinflammation [119].

### 5.4. Placental Dysfunction

Placental insufficiency in ZIKV-infected pregnant women can play a role in the impairment of neurogenesis, which can manifest as microcephaly. ZIKV infection of the placenta activates the immune response that culminates in the inflammation of the placenta and subsequent dysfunction of the placenta [26]. Placental insufficiency is associated with reduced blood supply to the fetus, which in turn, can have an adverse effect on NPCs [26]. Hirsch and coworkers [129] suggested that ZIKV infection can cause the inflammation and dysfunction of the placenta. The dysfunction of the placenta is associated with a reduction in blood flow to the fetus has been shown to cause restricted growth of the fetus and impaired neurodevelopment of the fetus [129]. Rabelo et al. [130] demonstrated that the ZIKV-infected placenta becomes inflamed and damaged. They suggested that the dysfunction of the placenta occurring in the setting of ZIKV infection has a subtle role to play in the disruption of the neurodevelopment of the fetus (Table 3) [130].

### 5.5. Congenital Zika Syndrome

ZIKV is responsible for CZS, a spectrum of congenital abnormalities, which is associated with maternal to fetal transmission [7,84]. Neural crest cells (NCCs) contribute to the development of the corneal stroma and endothelium, uveal stroma, sclera, and trabecular meshwork [120,121]. In addition to playing a crucial role in the developmental stages of the embryo, neural crest cells have a role to play in the closure of the optic fissure. The migration, proliferation, and differentiation of neural crest cells that occur during the embryonic developmental stages are necessary to generate the cells required to make up the various tissues of the eye. A disruption of this process can result in the impaired development of the various tissues of the anterior eye [120]. It is important to note that neural crest cells (NCCs) are susceptible to ZIKV infection since they express AXL. ZIKV utilizes the AXL expressed on neural crest cells to infect and replicate in these cells, resulting in loss of neural crest cells [100]. Additionally, ZIKV infection of the neural crest cells during the developmental stages of the fetus causes abnormal differentiation of these cells. This can result in the impaired development of the anterior segment of the eye, which manifests as microphthalmia (Table 3) [106,120,124,125]. The loss of NCCs associated with ZIKV infection can contribute to the disruption of the formation of the optic fissure, and failure to close the optic fissure during the developmental stages of the embryonic eye can result in the formation of iris coloboma (Table 3) [120,126]. Additionally, the failure of the optic fissure closure can cause abnormal development of the lens zonules [120]. ZIKV infection of neural crest cells results in abnormal proliferation and differentiation of ZIKV-infected NCCs into cells required for the normal development of the cornea [120]. Corneal ectasia due to reduced corneal stroma thickness (Table 3) [83] arises secondarily to defective morphogenesis of the cornea, involving ZIKV-infected neural crest cells [83,120]. Exposure of the fetus to ZIKV during the early developmental stages of the optic cup is likely to allow ZIKV access to the intraocular compartment [106]. ZIKV infection of neural crest cells during the developmental stages of the fetus can lead to the abnormal morphogenesis of the trabecular meshwork, which results in the abnormal differentiation of the trabecular meshwork cells [100,122,123]. This manifests as congenital glaucoma (Table 3) [93]. It is of note that ZIKV infects mesenchymal stem cells derived from neural crest cells during embryonic development, which results in the impaired proliferation and differentiation of cells required for the crystalline lens with the subsequent development of congenital cataracts (Table 3) [30,100,121,127,128]. Furthermore, the depletion of neurons during the developmental stages of the ZIKV-infected fetus can cause the attenuation of ganglion cells, which manifests as a thinning of the retinal ganglion cell layer [103,140]. It is important to note that retinal neural stem cells are susceptible to ZIKV infection, and as such, ZIKV-infected NSCs can undergo apoptosis with consequential reduction in axonal development (Table 3) [106].

### 5.6. Acute ZIKV Infection

During a blood meal by the Aedes mosquito, the virus is deposited within the skin where it gains access to the epidermal keratinocytes, dermal fibroblasts, macrophages, and dendritic cells [109]. ZIKV infects and replicates in epidermal keratinocytes and dermal fibroblasts. ZIKV in the infected epidermal keratinocytes and dermal fibroblasts are released from these lysed cells. The infection of these cells is associated with release of proinflammatory cytokines and chemokines that promote the influx of monocytes and monocyte-derived dendritic cells that are vulnerable to infection by ZIKV [109]. ZIKV, along with the infected dendritic cells and monocytes, is spread through the lymphatics to the regional lymph node. Lymphatic spread is followed by acute viremia characterized by the hematogenous spread of ZIKV to the mononuclear phagocyte system, where the replication of ZIKV occurs in hepatocytes [141] and splenocytes [142] to yield a high viral load. Following replication in the liver and spleen, ZIKV spreads to other organs, such as the brain and eyes [109]. The hematogenous spread of the virus to the eye results in a buildup of the ZIKV RNA load and the consequential persistence of virus in the eye, particularly in immune-privileged sites of the eye [143]. The infected dendritic cells and monocytes play a role in the hematogenous spread of ZIKV during the acute viremic phase to other sites, including the eyes [109]. There is an incubation period of 3–10 days with patients often complaining of mild fever and headache. ZIKV is usually associated with skin rash and conjunctivitis. It is of note that conjunctivitis, anterior uveitis with or without ocular hypertension, chorioretinitis, and maculopathy are commonly seen in patients with acute ZIKV infection (Table 3) [6,140].

#### 5.6.1. Retina

ZIKV-infected RPE cells and retinal vascular endothelial cells are blood–retinal barrier cells that serve as reservoirs for ZIKV and promote ZIKV dissemination. ZIKV-infected blood-retinal barrier cells produce TNFα, IL-6, IL-1β, CCL5, CXCL10, IFNα, and IFNβ [131]. ZIKV-induced chorioretinal atrophy is most likely due to immune-mediated inflammation directed at ZIKV-infected choroidal and retinal cells, which manifest as chorioretinal atrophy and the mottling of the RPE [131]. ZIKV-induced chorioretinitis is due to the intense replication of ZIKV and immune-mediated inflammation in response to replicating ZIKV in retinal cells of the blood–retinal barrier, particularly the RPE cells [132]. The inflammation causes damage to outer blood–retinal barrier cells, along with the adjacent choroid. The ZIKV-induced chorioretinitis results in the mottling and atrophy of RPE (Table 3) [131,132]. RPE pigmentation is due to melanosome, and a significant reduction in the biogenesis of melanosome causes a reduction in RPE pigmentation. ZIKV infection of RPE cells is associated with the modification of the pigmentation of RPE cells and this manifests as macular pigment mottling [105].

#### 5.6.2. Cornea 

ZIKV-infected corneal epithelial cells express TLR3, RIG-I, and MDA5 to facilitate the generation of type I and III interferons that mediate antiviral response [133,137]. Singh and coworkers [133] demonstrated that ZIKV-infected corneal epithelial cells support ZIKV replication as well as promote ZIKV persistence in the cornea. Because ZIKV can infect and replicate in corneal epithelial cells, the cornea can serve as a reservoir for ZIKV. As such, viral persistence in the cornea can pose a risk of ocular transmission [133]. It has been reported that ZIKV-infected corneal epithelial cells can secrete proinflammatory cytokines (TNFα and IL-1β) and chemokines (CCL5 and CXCL10) that promote the recruitment of effector immune cells to the cornea. These cells cause inflammation and damage of the corneal epithelium, which manifests as keratitis (Table 3) [133]. As such, ZIKV can cause keratitis.

#### 5.6.3. Trabecular Meshwork

The trabecular meshwork located in the iridocorneal angle consists of four major cell types, i.e., macrophages, fibroblasts, endothelial cells, and smooth muscle cells [144]. The endothelial cells are responsible for maintaining the patency of the passageway through which aqueous flows out of the anterior chamber, whereas macrophages are responsible for phagocytosis and immune responses. Fibroblasts produce the extracellular matrix, and smooth muscle cells are responsible for the contractile tone [144]. Following ZIKV infection, there is hematogenous spread of ZIKV to the iris and ciliary body. In the iris and ciliary body, ZIKV can infect and replicate in blood-aqueous barrier cells (vascular endothelial cells of the iris and nonpigmented ciliary epithelial cells), which can result in the breakdown of the blood–aqueous barrier, and subsequent access of ZIKV to the aqueous humor [134]. ZIKV in the aqueous humor reaches the trabecular meshwork, where it infects the vulnerable trabecular meshwork cells. This induces an antiviral response and innate immune response, with the ZIKV-infected trabecular meshwork cells secreting cytokines (TNFα, IL-6, IL-1β, IL-12, IFNα, and IFNβ) and chemokines (CCL5 and CXCL10) that promote the recruitment of effector immune cells, such as Th1 cells and CD8+T cells, to the trabecular meshwork. Th1 cells secrete TNFα and IFNγ that induce cytokine-mediated inflammatory response in the trabecular meshwork, which manifests as trabeculitis (Table 3) [134]. The inflammation of the trabecular meshwork can destroy trabecular meshwork cells, resulting in a dysfunctional passageway that restricts the outflow of aqueous humor. This leads to an elevation in intraocular pressure, which, in turn, can cause glaucomatous optic neuropathy characterized by the destruction of retinal ganglion cells and optic nerve [134].

#### 5.6.4. Conjunctiva

Epithelial cells of the conjunctiva express RIG-I, MDA5, and TLR3. Thus, ZIKV infection of conjunctival epithelial cells results in the induction of both an antiviral immune response and immune-mediated inflammatory response, which manifests as an inflammation of the conjunctiva (Table 3) [135]. It has been reported that nonpurulent conjunctivitis is usually associated with dilated conjunctival vasculature, which usually occurs during the acute viremic phase of ZIKV infection. Vascular endothelial cells are prone to infection by ZIKV, which results in an upregulation of ICAM-1 on vascular endothelial cells of the conjunctiva. ICAM-1 induces vascular dilatation, which manifest as hyperemia and is characteristic of ZIKV-induced nonpurulent conjunctivitis [11,106]. It has been suggested that nonpurulent conjunctivitis in ZIKV infection is likely due to the immune response to ZIKV in the conjunctiva. ICAM-1 also induces vasopermeability that facilitates the transmigration of effector immune cells into the conjunctival to promote inflammation of the conjunctiva [11].

#### 5.6.5. Anterior Uvea

ZIKV-induced anterior uveitis occurs through a combination of virus-induced cytopathic effects of the infected iris pigment epithelial cells and immune-mediated inflammation triggered by innate and adaptive immune responses to the ZIKV-infected iris pigment epithelial cells. Th1 cells recruited to the infected tissue secrete TNFα and IFNγ, which promote the cytokine-mediated inflammation of the infected tissue. CD8+T cells secrete perforin and granzyme that induce the direct cytotoxic destruction of ZIKV-infected cells [137,138]. Iris pigment epithelial cells express TLR3 [136]. Iris pigment epithelial cells are susceptible to infection by ZIKV. The replication of ZIKV in the iris pigment epithelial layer triggers an intense immune-mediated inflammatory response, resulting in the development of anterior uveitis (Table 3) [137]. It is important to note that type I and III interferons are expressed by ZIKV-infected human iris pigment epithelial cells. Furthermore, ZIKV-associated anterior uveitis can occur when ZIKV-infected myeloid cells in the blood stream gain access to the anterior uvea during acute ZIKV infection. The permeability of the ZIKV-infected iris vessel facilitates the influx of both ZIKV-infected myeloid cells and effector T cells that contribute to the inflammatory process in the anterior uvea [137]. The ocular hypertension in ZIKV-associated anterior uveitis is due to trabeculitis, clogging of the trabecular meshwork by inflammatory debris and effector immune cells, hyperviscosity of the aqueous humor, and the influx of plasma into the anterior chamber through dilated and permeable vessels in the inflamed anterior uvea [137,139].

## 6. Conclusions

Ocular findings associated with congenital ZIKV syndrome include iris coloboma, microphthalmia, optic disc pallor, chorioretinal atrophy, lens subluxation, cataract, glaucoma, strabismus, retinal pigment epithelial mottling, maculopathy, abnormal retinal vessels, and pupillary abnormalities [5,6]. It is important to rule out other causes of congenital infections. The differential diagnosis of ZIKV CZS includes congenital infections caused by cytomegalovirus, (CMV), herpes simplex virus (HSV), toxoplasmosis gondii, rubella virus, and syphilis [88]. In ZIKV-endemic areas, infants who present with presumed congenital ocular disease related to infection should undergo testing to rule out ZIKV seropositivity, particularly if their mothers reported having signs and symptoms of ZIKV during pregnancy [61]. Nonpurulent conjunctivitis with bilateral non-granulomatous anterior uveitis, chorioretinitis, trabeculitis, keratitis, and maculopathy are ocular findings seen in patients with acute ZIKV infection [6,140].

There are currently no specific FDA approved medicines for treating ZIKV infection; however, specific pharmacotherapy is required to treat the ocular manifestations of ZIKV infections [16,145,146]. Anterior segment conditions, such as conjunctivitis and keratitis, are treated with topical antimicrobials and palliative care. Anterior uveitis or iridocyclitis is treated with a combination of topical steroids and cycloplegics. When IOP is elevated in these cases, additional topical antiglaucoma medications, such as beta blockers, carbonic anhydrase inhibitors, and alpha-2 agonists, are also utilized. Prostaglandins may also be used but are not first-line due to the inflammatory nature of the IOP elevation [147]. Treatment for congenital glaucoma is surgical and includes procedures such as goniotomy, cycloablation, trabeculotomy, and the use of drainage devices to lower IOP and prevent damage to the optic nerve [148]. Posterior segment manifestations of ZIKV are largely untreated. For cases in which a choroiditis develops, systemic corticosteroids or immunomodulators may be used [149]. Because there is no approved specific therapy for ZIKV infection, preventive strategies, such as reducing the risk associated with both vector and non-vector transmission, are necessary. Draining mosquito breeding grounds and using insecticides are important measures to prevent ZIKV infection acquired through mosquito bites [16,145,146]. Because of the neuro-ophthalmic and ocular complications associated with ZIKV infection, it is imperative to develop and utilize antiviral agents that target the ZIKV proteins that are responsible for immune evasion and viral replication [150]. It is of note that EDIII is an important structural protein that is responsible for mediating the attachment of the virus to receptors on permissive host cells, and, as such, developing antiviral therapies that could attenuate the ability of the virus to engage surface receptors on permissive cells should be considered one of the most important antiviral strategies. Furthermore, vaccine platforms that boost the generation of cell-mediated immunity against nonstructural proteins that are responsible for immune evasion and viral replication should be the focus of continued and future research.

## Figures and Tables

**Table 1:** Ocular findings documented in acute ZIKV infection.

Anterior Segment	Neuro-Ophthalmic	Posterior Segment
Nonpurulent conjunctivitis [62]. Anterior uveitis (bilateral, nongranulomatous iridocyclitis with or without elevated intraocular pressure [57,64].	Papilledema [66]. Ophthalmoplegia [5,66]. Ocular flutter [67].	Maculopathy with outer retinal layer and RPE disruption [57,61]. Multifocal choroiditis [65].

**Table 2:** Ocular findings documented in CZS.

Anterior Segment	Neuro-Ophthalmic	Posterior Segment
Lens subluxation [61,63]. Cataract [63,76,78]. Intraocular calcifications [63,76,78]. Congenital glaucoma [80] Microphthalmia [81,82]. Corneal ectasia [83]. Iris coloboma [63,84].	Strabismus [79,85]. Horizontal nystagmus [79,85]. Exophoria/esophoria [79,85]. Loss of pupillary response [79,85]. Disc hypoplasia [79,85]. Disc pallor [79,85]. Enlarged cup-to-disc ratio [79,85].	Macular pigment mottling [5,79]. Chorioretinal atrophy either macular, paramacular, or peripheral [5,79]. Retinal hemorrhage [86]. Vascular tortuosity [85]. Early termination of retinal vasculature [85]. Washed out peripheral retina with hypoluscent spot [85]. Photoreceptor, RPE thinning with pigment loss, and choroidal thinning [87,88].

**Table 3:** Pathological mechanisms and manifestations of ZIKV infection.

Primary Targets	Receptors	Mechanisms	Outcomes
Neural progenitor cells	AXL [99].	Apoptosis of ZIKV-infected NPCs. Reduced proliferation of NPCs. Premature differentiation of NPCs [113,115].	Microcephaly [115].
Neural crest cells	AXL [100].	ZIKV infection of NCCs during the developmental stages of the fetus causes abnormal migration, proliferation, and differentiation of NCCs [100,120]. The loss of NCCs associated with ZIKV infection can contribute to the disruption of the formation of the optic fissure, resulting in failure to close the optic fissure [86,120]. ZIKV infection of NCCs will result in abnormal proliferation and differentiation of cells required for the normal development of the cornea [121]. ZIKV infection of NCCs during the developmental stages of the fetus can lead to abnormal morphogenesis of the trabecular meshwork [100,122,123].	Microphthalmia [106,120,124,125]. Iris coloboma [7,84,120,126]. Corneal ectasia [83]. Congenital glaucoma [93,100,122,123].
Mesenchymal stem cells	AXL [101].	ZIKV infects mesenchymal stem cells, resulting in impaired proliferation and differentiation of cells required for the development of the crystalline lens [30,100,121,127,128].	Congenital cataract [30,100,121,127,128].
Placental endothelial cells and trophoblasts	TIM-1, AXL, TYRO3 [27].	Destruction of ZIKV-infected placenta [129,130]. Compromised maternal-fetal interface [129,130].	Placental insufficiency causes restricted growth of the fetus and disruption of neurodevelopment of the fetus [129]. Facilitate access of ZIKV to the fetus [129,130].
Blood retinal barrier cells (Retinal vascular endothelial cells and retinal pigment epithelial cells)	AXL, TYRO3, TIM-1, RIG-I/MDA5, TLR3 [20].	ZIKV infected BRB cells induce inflammation that damages the BRB and facilitate influx of effector immune cells into the retina [131,132].	Chorioretinitis, macular pigment mottling, chorioretinal atrophy, and maculopathy [131,132].
Cornea epithelial cells	TLR3, RIG-I, MDA5 [133].	ZIKV-infected corneal epithelium induces antiviral response and immune-mediated inflammation [133].	Keratitis [133].
Trabecular meshwork cells	RIG-I, TLR3 [134].	ZIKV-infected trabecular meshwork cells secrete cytokines and chemokines that promote inflammation via the recruitment of effector immune cells such as Th1 cells to the trabecular meshwork [134].	Trabeculitis [134].
Conjunctival epithelial cells	RIG-I/MDA5, TLR3 [135].	ZIKV-infected conjunctival epithelial cells induce an immune-mediated inflammatory response [135].	Nonpurulent conjunctivitis [135].
Iris pigment epithelium	TLR3 [136].	Immune-mediated inflammation triggered in response to ZIKV-infected iris pigment epithelium. ZIKV-infected blood aqueous barrier (BAB) cells induce inflammation that damages the BAB and facilitate influx of effector immune cells into the anterior uvea. Influx of ZIKV-infected monocytes acting as Trojan horses [137,138,139].	Anterior uveitis with or without raised intraocular pressure [137,138,139].

## Data Availability

No new data were created or analyzed in this study. Data sharing is not applicable to this article.

## References

[1] Song B.H., Yun S.I., Woolley M., Lee Y.M. (2017). Zika virus: History, epidemiology, transmission, and clinical presentation. J. Neuroimmunol..

[2] Dick G.W. (1952). Zika virus. II. Pathogenicity and physical properties. Trans. R. Soc. Trop. Med. Hyg..

[3] Dick G.W., Kitchen S.F., Haddow A.J. (1952). Zika virus. I. Isolations and serological specificity. Trans. R. Soc. Trop. Med. Hyg..

[4] Culshaw A., Mongkolsapaya J., Screaton G. (2018). The immunology of Zika Virus. F1000Research.

[5] Marquezan M.C., Ventura C.V., Sheffield J.S., Golden W.C., Omiadze R., Belfort R., May W. (2018). Ocular effects of Zika virus-a review. Surv. Ophthalmol..

[6] Merle H., Donnio A., Jean-Charles A., Guyomarch J., Hage R., Najioullah F., Cesaire R., Cabie A. (2018). Ocular manifestations of emerging arboviruses: Dengue fever, Chikungunya, Zika virus, West Nile virus, and yellow fever. J. Français Ophtalmol..

[7] Gregory C.J., Oduyebo T., Brault A.C., Brooks J.T., Chung K.W., Hills S., Kuehnert M.J., Mead P., Meaney-Delman D., Rabe I. (2017). Modes of Transmission of Zika Virus. J. Infect. Dis..

[8] Petersen L.R., Jamieson D.J., Powers A.M., Honein M.A. (2016). Zika Virus. N. Engl. J. Med..

[9] Younger D.S. (2016). Epidemiology of Zika Virus. Neurol. Clin..

[10] Beaver J.T., Lelutiu N., Habib R., Skountzou I. (2018). Evolution of Two Major Zika Virus Lineages: Implications for Pathology, Immune Response, and Vaccine Development. Front. Immunol..

[11] Tan J.J.L., Balne P.K., Leo Y.S., Tong L., Ng L.F.P., Agrawal R. (2017). Persistence of Zika virus in conjunctival fluid of convalescence patients. Sci. Rep..

[12] Swaminathan S., Schlaberg R., Lewis J., Hanson K.E., Couturier M.R. (2016). Fatal Zika Virus Infection with Secondary Nonsexual Transmission. N. Engl. J. Med..

[13] Barreto-Vieira D.F., Jacome F.C., da Silva M.A.N., Caldas G.C., de Filippis A.M.B., de Sequeira P.C., de Souza E.M., Andrade A.A., Manso P.P.A., Trindade G.F. (2017). Structural investigation of C6/36 and Vero cell cultures infected with a Brazilian Zika virus. PLoS ONE.

[14] Zhao H., Fernandez E., Dowd K.A., Speer S.D., Platt D.J., Gorman M.J., Govero J., Nelson C.A., Pierson T.C., Diamond M.S. (2016). Structural Basis of Zika Virus-Specific Antibody Protection. Cell.

[15] Shankar A., Patil A.A., Skariyachan S. (2017). Recent Perspectives on Genome, Transmission, Clinical Manifestation, Diagnosis, Therapeutic Strategies, Vaccine Developments, and Challenges of Zika Virus Research. Front. Microbiol..

[16] Atif M., Azeem M., Sarwar M.R., Bashir A. (2016). Zika virus disease: A current review of the literature. Infection.

[17] Sironi M., Forni D., Clerici M., Cagliani R. (2016). Nonstructural Proteins Are Preferential Positive Selection Targets in Zika Virus and Related Flaviviruses. PLoS Negl. Trop. Dis..

[18] Kostyuchenko V.A., Lim E.X., Zhang S., Fibriansah G., Ng T.S., Ooi J.S., Shi J., Lok S.M. (2016). Structure of the thermally stable Zika virus. Nature.

[19] Kim J.A., Seong R.K., Son S.W., Shin O.S. (2019). Insights into ZIKV-Mediated Innate Immune Responses in Human Dermal Fibroblasts and Epidermal Keratinocytes. J. Investig. Dermatol..

[20] Lee I., Bos S., Li G., Wang S., Gadea G., Despres P., Zhao R.Y. (2018). Probing Molecular Insights into Zika Virus–Host Interactions. Viruses.

[21] Robbiani D.F., Bozzacco L., Keeffe J.R., Khouri R., Olsen P.C., Gazumyan A., Schaefer-Babajew D., Avila-Rios S., Nogueira L., Patel R. (2017). Recurrent Potent Human Neutralizing Antibodies to Zika Virus in Brazil and Mexico. Cell.

[22] Valente A.P., Moraes A.H. (2019). Zika virus proteins at an atomic scale: How does structural biology help us to understand and develop vaccines and drugs against Zika virus infection?. J. Venom. Anim. Toxins Incl. Trop. Dis..

[23] Luo D., Vasudevan S.G., Lescar J. (2015). The flavivirus NS2B-NS3 protease-helicase as a target for antiviral drug development. Antivir. Res..

[24] Lee J.K., Shin O.S. (2019). Advances in Zika Virus(-)Host Cell Interaction: Current Knowledge and Future Perspectives. Int. J. Mol. Sci..

[25] Da Silva M.H.M., Moises R.N.C., Alves B.E.B., Pereira H.W.B., de Paiva A.A.P., Morais I.C., Nascimento Y.M., Monteiro J.D., de Souto J.T., Nascimento M.S.L. (2019). Innate immune response in patients with acute Zika virus infection. Med. Microbiol. Immunol..

[26] Wen Z., Song H., Ming G.L. (2017). How does Zika virus cause microcephaly?. Genes Dev..

[27] Routhu N.K., Byrareddy S.N. (2017). Host-Virus Interaction of ZIKA Virus in Modulating Disease Pathogenesis. J. Neuroimmune Pharmacol..

[28] Sirohi D., Kuhn R.J. (2017). Zika Virus Structure, Maturation, and Receptors. J. Infect. Dis..

[29] Chigbu D.I., Jain P., Crumley B.L., Patel D., Khan Z.K. (2019). Human T cell leukemia virus type 1 and Zika virus: Tale of two reemerging viruses with neuropathological sequelae of public health concern. J. Neurovirol..

[30] Beys-da-Silva W.O., Rosa R.L., Santi L., Berger M., Park S.K., Campos A.R., Terraciano P., Varela A.P.M., Teixeira T.F., Roehe P.M. (2019). Zika Virus Infection of Human Mesenchymal Stem Cells Promotes Differential Expression of Proteins Linked to Several Neurological Diseases. Mol. Neurobiol..

[31] Hamel R., Dejarnac O., Wichit S., Ekchariyawat P., Neyret A., Luplertlop N., Perera-Lecoin M., Surasombatpattana P., Talignani L., Thomas F. (2015). Biology of Zika Virus Infection in Human Skin Cells. J. Virol..

[32] Byrne A.B., Talarico L.B. (2021). Role of the complement system in antibody-dependent enhancement of flavivirus infections. Int. J. Infect. Dis..

[33] Merle N.S., Noe R., Halbwachs-Mecarelli L., Fremeaux-Bacchi V., Roumenina L.T. (2015). Complement System Part II: Role in Immunity. Front. Immunol..

[34] Chung K.M., Liszewski M.K., Nybakken G., Davis A.E., Townsend R.R., Fremont D.H., Atkinson J.P., Diamond M.S. (2006). West Nile virus nonstructural protein NS1 inhibits complement activation by binding the regulatory protein factor H. Proc. Natl. Acad. Sci. USA.

[35] Malekshahi Z., Schiela B., Bernklau S., Banki Z., Wurzner R., Stoiber H. (2020). Interference of the Zika Virus E-Protein With the Membrane Attack Complex of the Complement System. Front. Immunol..

[36] Ma J., Ketkar H., Geng T., Lo E., Wang L., Xi J., Sun Q., Zhu Z., Cui Y., Yang L. (2018). Zika Virus Non-structural Protein 4A Blocks the RLR-MAVS Signaling. Front. Microbiol..

[37] Liu S., Cai X., Wu J., Cong Q., Chen X., Li T., Du F., Ren J., Wu Y.T., Grishin N.V. (2015). Phosphorylation of innate immune adaptor proteins MAVS, STING, and TRIF induces IRF3 activation. Science.

[38] Sun L., Liu S., Chen Z.J. (2010). SnapShot: Pathways of antiviral innate immunity. Cell.

[39] Seth R.B., Sun L., Chen Z.J. (2006). Antiviral innate immunity pathways. Cell Res..

[40] Vercammen E., Staal J., Beyaert R. (2008). Sensing of viral infection and activation of innate immunity by toll-like receptor 3. Clin. Microbiol. Rev..

[41] Hacker H., Redecke V., Blagoev B., Kratchmarova I., Hsu L.C., Wang G.G., Kamps M.P., Raz E., Wagner H., Hacker G. (2006). Specificity in Toll-like receptor signalling through distinct effector functions of TRAF3 and TRAF6. Nature.

[42] Platanias L.C. (2005). Mechanisms of type-I- and type-II-interferon-mediated signalling. Nat. Rev. Immunol..

[43] Ivashkiv L.B., Donlin L.T. (2014). Regulation of type I interferon responses. Nat. Rev. Immunol..

[44] Bjorkstrom N.K., Strunz B., Ljunggren H.G. (2022). Natural killer cells in antiviral immunity. Nat. Rev. Immunol..

[45] Marongiu L., Valache M., Facchini F.A., Granucci F. (2021). How dendritic cells sense and respond to viral infections. Clin. Sci..

[46] Swain S.L., McKinstry K.K., Strutt T.M. (2012). Expanding roles for CD4(+) T cells in immunity to viruses. Nat. Rev. Immunol..

[47] Hassert M., Wolf K.J., Schwetye K.E., DiPaolo R.J., Brien J.D., Pinto A.K. (2018). CD4+T cells mediate protection against Zika associated severe disease in a mouse model of infection. PLoS Pathog..

[48] Tonnerre P., Melgaco J.G., Torres-Cornejo A., Pinto M.A., Yue C., Blumel J., de Sousa P.S.F., de Mello V.D.M., Moran J., de Filippis A.M.B. (2020). Evolution of the innate and adaptive immune response in women with acute Zika virus infection. Nat. Microbiol..

[49] Laidlaw B.J., Craft J.E., Kaech S.M. (2016). The multifaceted role of CD4(+) T cells in CD8(+) T cell memory. Nat. Rev. Immunol..

[50] Elong Ngono A., Vizcarra E.A., Tang W.W., Sheets N., Joo Y., Kim K., Gorman M.J., Diamond M.S., Shresta S. (2017). Mapping and Role of the CD8(+) T Cell Response During Primary Zika Virus Infection in Mice. Cell Host Microbe.

[51] Priyamvada L., Suthar M.S., Ahmed R., Wrammert J. (2017). Humoral Immune Responses Against Zika Virus Infection and the Importance of Preexisting Flavivirus Immunity. J. Infect. Dis..

[52] Lai L., Rouphael N., Xu Y., Natrajan M.S., Beck A., Hart M., Feldhammer M., Feldpausch A., Hill C., Wu H. (2018). Innate, T-, and B-Cell Responses in Acute Human Zika Patients. Clin. Infect. Dis..

[53] Screaton G., Mongkolsapaya J., Yacoub S., Roberts C. (2015). New insights into the immunopathology and control of dengue virus infection. Nat. Rev. Immunol..

[54] Cabral-Miranda G., Lim S.M., Mohsen M.O., Pobelov I.V., Roesti E.S., Heath M.D., Skinner M.A., Kramer M.F., Martina B.E.E., Bachmann M.F. (2019). Zika Virus-Derived E-DIII Protein Displayed on Immunologically Optimized VLPs Induces Neutralizing Antibodies without Causing Enhancement of Dengue Virus Infection. Vaccines.

[55] Stettler K., Beltramello M., Espinosa D.A., Graham V., Cassotta A., Bianchi S., Vanzetta F., Minola A., Jaconi S., Mele F. (2016). Specificity, cross-reactivity, and function of antibodies elicited by Zika virus infection. Science.

[56] Agrawal R., Oo H.H., Balne P.K., Ng L., Tong L., Leo Y.S. (2018). Zika Virus and the Eye. Ocul. Immunol. Inflamm..

[57] Furtado J.M., Esposito D.L., Klein T.M., Teixeira-Pinto T., da Fonseca B.A. (2016). Uveitis Associated with Zika Virus Infection. N. Engl. J. Med..

[58] Troumani Y., Touhami S., Jackson T.L., Ventura C.V., Stanescu-Segall D.M., Errera M.H., Rousset D., Bodaghi B., Cartry G., David T. (2021). Association of Anterior Uveitis With Acute Zika Virus Infection in Adults. JAMA Ophthalmol..

[59] Kodati S., Palmore T.N., Spellman F.A., Cunningham D., Weistrop B., Sen H.N. (2017). Bilateral posterior uveitis associated with Zika virus infection. Lancet.

[60] Parke D.W., Almeida D.R., Albini T.A., Ventura C.V., Berrocal A.M., Mittra R.A. (2016). Serologically Confirmed Zika-Related Unilateral Acute Maculopathy in an Adult. Ophthalmology.

[61] De Paula Freitas B., Ventura C.V., Maia M., Belfort R. (2017). Zika virus and the eye. Curr. Opin. Ophthalmol..

[62] Buathong R., Hermann L., Thaisomboonsuk B., Rutvisuttinunt W., Klungthong C., Chinnawirotpisan P., Manasatienkij W., Nisalak A., Fernandez S., Yoon I.K. (2015). Detection of Zika Virus Infection in Thailand, 2012–2014. Am. J. Trop. Med. Hyg..

[63] De Paula Freitas B., de Oliveira Dias J.R., Prazeres J., Sacramento G.A., Ko A.I., Maia M., Belfort R. (2016). Ocular Findings in Infants With Microcephaly Associated With Presumed Zika Virus Congenital Infection in Salvador, Brazil. JAMA Ophthalmol..

[64] Fontes B.M. (2016). Zika virus-related hypertensive iridocyclitis. Arq. Bras. Oftalmol..

[65] Jimenez P., Kestelman E., Kestelman B., Vizzoni A.G., Cerbino-Neto J., Curi A.L.L. (2020). Multifocal Choroiditis Secondary to Acute Zika Virus Infection. Ocul. Immunol. Inflamm..

[66] Man B.L. (2014). Total internal and external ophthalmoplegia as presenting symptoms of Miller Fisher syndrome. BMJ Case Rep..

[67] Karam E., Giraldo J., Rodriguez F., Hernandez-Pereira C.E., Rodriguez-Morales A.J., Blohm G.M., Paniz-Mondolfi A.E. (2017). Ocular flutter following Zika virus infection. J. Neurovirol..

[68] Duffy M.R., Chen T.H., Hancock W.T., Powers A.M., Kool J.L., Lanciotti R.S., Pretrick M., Marfel M., Holzbauer S., Dubray C. (2009). Zika virus outbreak on Yap Island, Federated States of Micronesia. N. Engl. J. Med..

[69] Parra B., Lizarazo J., Jimenez-Arango J.A., Zea-Vera A.F., Gonzalez-Manrique G., Vargas J., Angarita J.A., Zuniga G., Lopez-Gonzalez R., Beltran C.L. (2016). Guillain-Barre Syndrome Associated with Zika Virus Infection in Colombia. N. Engl. J. Med..

[70] Acosta-Ampudia Y., Monsalve D.M., Castillo-Medina L.F., Rodriguez Y., Pacheco Y., Halstead S., Willison H.J., Anaya J.M., Ramirez-Santana C. (2018). Autoimmune Neurological Conditions Associated With Zika Virus Infection. Front. Mol. Neurosci..

[71] White M.K., Wollebo H.S., David Beckham J., Tyler K.L., Khalili K. (2016). Zika virus: An emergent neuropathological agent. Ann. Neurol..

[72] Lemos J., Eggenberger E. (2013). Saccadic intrusions: Review and update. Curr. Opin. Neurol..

[73] Wiest G., Safoschnik G., Schnaberth G., Mueller C. (1997). Ocular flutter and truncal ataxia may be associated with enterovirus infection. J. Neurol..

[74] Do Rosario M.S., Giovanetti M., de Jesus P.A.P., Farias D.S., Faria N.R., de Lima C.P.S., da Silva S.P., Nunes M.R., Alcantara L.C.J., de Siqueira I.C. (2018). Opsoclonus-myoclonus-ataxia syndrome associated with chikungunya and dengue virus co-infection. Int. J. Infect. Dis..

[75] Ventura C.V., Maia M., Travassos S.B., Martins T.T., Patriota F., Nunes M.E., Agra C., Torres V.L., van der Linden V., Ramos R.C. (2016). Risk Factors Associated With the Ophthalmoscopic Findings Identified in Infants With Presumed Zika Virus Congenital Infection. JAMA Ophthalmol..

[76] Yasri S., Wiwanitkit V. (2019). Glaucoma in Congenital Zika Syndrome. J. Glaucoma.

[77] Leyser M., Nascimento O.J.M. (2017). Congenital Zika Virus Infection: Beyond Neonatal Microcephaly. JAMA Neurol..

[78] Cofre F. (2016). Zika virus intrauterine infection causes fetal brain abnormality and microcephaly: Tip of the iceberg?. Rev. Chilena. Infectol..

[79] Ventura C.V., Maia M., Bravo-Filho V., Gois A.L., Belfort R. (2016). Zika virus in Brazil and macular atrophy in a child with microcephaly. Lancet.

[80] Yepez J.B., Murati F.A., Pettito M., Penaranda C.F., de Yepez J., Maestre G., Arevalo J.F., Johns Hopkins Zika C. (2017). Ophthalmic Manifestations of Congenital Zika Syndrome in Colombia and Venezuela. JAMA Ophthalmol..

[81] Schuler-Faccini L., Ribeiro E.M., Feitosa I.M., Horovitz D.D., Cavalcanti D.P., Pessoa A., Doriqui M.J., Neri J.I., Neto J.M., Wanderley H.Y. (2016). Possible Association Between Zika Virus Infection and Microcephaly—Brazil, 2015. MMWR. Morb. Mortal. Wkly. Rep..

[82] Sahiner F., Sig A.K., Savasci U., Tekin K., Akay F. (2017). Zika Virus-associated Ocular and Neurologic Disorders: The Emergence of New Evidence. Pediatr. Infect. Dis. J..

[83] Vasconcelos G.C., Macedo Pereira C.M., Toledo de Paula C.H., de Souza Haueisen Barbosa P., Machado de Souza D., Coelho L.M. (2019). Corneal ectasia and high ametropia in an infant with microcephaly associated with presumed Zika virus congenital infection: New ocular findings. J. AAPOS.

[84] De Paula Freitas B., Zin A., Ko A., Maia M., Ventura C.V., Belfort R. (2017). Anterior-Segment Ocular Findings and Microphthalmia in Congenital Zika Syndrome. Ophthalmology.

[85] Ventura C.V., Maia M., Ventura B.V., Linden V.V., Araujo E.B., Ramos R.C., Rocha M.A., Carvalho M.D., Belfort R., Ventura L.O. (2016). Ophthalmological findings in infants with microcephaly and presumable intra-uterus Zika virus infection. Arq. Bras. Oftalmol..

[86] Zin A.A., Tsui I., Rossetto J., Vasconcelos Z., Adachi K., Valderramos S., Halai U.A., Pone M., Pone S.M., Silveira Filho J.C.B. (2017). Screening Criteria for Ophthalmic Manifestations of Congenital Zika Virus Infection. JAMA Pediatr..

[87] Ventura C.V., Ventura L.O., Bravo-Filho V., Martins T.T., Berrocal A.M., Gois A.L., de Oliveira Dias J.R., Araujo L., Escariao P., van der Linden V. (2016). Optical Coherence Tomography of Retinal Lesions in Infants With Congenital Zika Syndrome. JAMA Ophthalmol..

[88] De Oliveira Dias J.R., Ventura C.V., de Paula Freitas B., Prazeres J., Ventura L.O., Bravo-Filho V., Aleman T., Ko A.I., Zin A., Belfort R. (2018). Zika and the Eye: Pieces of a Puzzle. Prog. Retin. Eye Res..

[89] Miranda H.A., Costa M.C., Frazao M.A.M., Simao N., Franchischini S., Moshfeghi D.M. (2016). Expanded Spectrum of Congenital Ocular Findings in Microcephaly with Presumed Zika Infection. Ophthalmology.

[90] Ventura C.V., Zin A., Paula Freitas B., Ventura L.O., Rocha C., Costa F., Nery N., De Senna T.C.R., Lopes Moreira M.E., Maia M. (2021). Ophthalmological manifestations in congenital Zika syndrome in 469 Brazilian children. J. AAPOS.

[91] Vercosa I., Carneiro P., Vercosa R., Girao R., Ribeiro E.M., Pessoa A., Almeida N.G., Vercosa P., Tartarella M.B. (2017). The visual system in infants with microcephaly related to presumed congenital Zika syndrome. J. AAPOS.

[92] De Oliveira Dias J.R., Ventura C.V., Borba P.D., de Paula Freitas B., Pierroti L.C., do Nascimento A.P., de Moraes N.S.B., Maia M., Belfort R. (2018). Infants with Congenital Zika Syndrome and Ocular Findings from Sao Paulo, Brazil: Spread of Infection. Retin. Cases Brief Rep..

[93] De Paula Freitas B., Ko A.I., Khouri R., Mayoral M., Henriques D.F., Maia M., Belfort R. (2017). Glaucoma and Congenital Zika Syndrome. Ophthalmology.

[94] Martins M.M., Medronho R.A., Cunha A. (2021). Zika virus in Brazil and worldwide: A narrative review. Paediatr. Int. Child Health.

[95] Maharajan M.K., Ranjan A., Chu J.F., Foo W.L., Chai Z.X., Lau E.Y., Ye H.M., Theam X.J., Lok Y.L. (2016). Zika Virus Infection: Current Concerns and Perspectives. Clin. Rev. Allergy Immunol..

[96] Sun X., Hua S., Chen H.R., Ouyang Z., Einkauf K., Tse S., Ard K., Ciaranello A., Yawetz S., Sax P. (2017). Transcriptional Changes during Naturally Acquired Zika Virus Infection Render Dendritic Cells Highly Conducive to Viral Replication. Cell Rep..

[97] Lugo-Villarino G., Troegeler A., Balboa L., Lastrucci C., Duval C., Mercier I., Benard A., Capilla F., Al Saati T., Poincloux R. (2018). The C-Type Lectin Receptor DC-SIGN Has an Anti-Inflammatory Role in Human M(IL-4) Macrophages in Response to Mycobacterium tuberculosis. Front. Immunol..

[98] Tsou W.I., Nguyen K.Q., Calarese D.A., Garforth S.J., Antes A.L., Smirnov S.V., Almo S.C., Birge R.B., Kotenko S.V. (2014). Receptor tyrosine kinases, TYRO3, AXL, and MER, demonstrate distinct patterns and complex regulation of ligand-induced activation. J. Biol. Chem.

[99] Nowakowski T.J., Pollen A.A., Di Lullo E., Sandoval-Espinosa C., Bershteyn M., Kriegstein A.R. (2016). Expression Analysis Highlights AXL as a Candidate Zika Virus Entry Receptor in Neural Stem Cells. Cell Stem Cell.

[100] Bayless N.L., Greenberg R.S., Swigut T., Wysocka J., Blish C.A. (2016). Zika Virus Infection Induces Cranial Neural Crest Cells to Produce Cytokines at Levels Detrimental for Neurogenesis. Cell Host Microbe.

[101] Wu G., Ma Z., Hu W., Wang D., Gong B., Fan C., Jiang S., Li T., Gao J., Yang Y. (2017). Molecular insights of Gas6/TAM in cancer development and therapy. Cell Death Dis..

[102] Roach T., Alcendor D.J. (2017). Zika virus infection of cellular components of the blood-retinal barriers: Implications for viral associated congenital ocular disease. J. Neuroinflamm..

[103] Zhao Z., Yang M., Azar S.R., Soong L., Weaver S.C., Sun J., Chen Y., Rossi S.L., Cai J. (2017). Viral Retinopathy in Experimental Models of Zika Infection. Investig. Ophthalmol. Vis. Sci..

[104] Vielle N.J., Zumkehr B., Garcia-Nicolas O., Blank F., Stojanov M., Musso D., Baud D., Summerfield A., Alves M.P. (2018). Silent infection of human dendritic cells by African and Asian strains of Zika virus. Sci. Rep..

[105] Simonin Y., Erkilic N., Damodar K., Cle M., Desmetz C., Bollore K., Taleb M., Torriano S., Barthelemy J., Dubois G. (2019). Zika virus induces strong inflammatory responses and impairs homeostasis and function of the human retinal pigment epithelium. eBioMedicine.

[106] Nelson B.R., Roby J.A., Dobyns W.B., Rajagopal L., Gale M., Adams Waldorf K.M. (2020). Immune Evasion Strategies Used by Zika Virus to Infect the Fetal Eye and Brain. Viral Immunol..

[107] Beaver J.T., Mills L.K., Swieboda D., Lelutiu N., Esser E.S., Antao O.Q., Scountzou E., Williams D.T., Papaioannou N., Littauer E.Q. (2020). Zika virus-induced neuro-ocular pathology in immunocompetent mice correlates with anti-ganglioside autoantibodies. Hum. Vaccines Immunother..

[108] Hoffmann S., He S., Ehren M., Ryan S.J., Wiedemann P., Hinton D.R. (2006). MMP-2 and MMP-9 secretion by rpe is stimulated by angiogenic molecules found in choroidal neovascular membranes. Retina.

[109] Young A.T.L., Moore R.B., Murray A.G., Mullen J.C., Lakey J.R.T. (2004). Assessment of Different Transfection Parameters in Efficiency Optimization. Cell Transpl..

[110] McDonald E.M., Anderson J., Wilusz J., Ebel G.D., Brault A.C. (2020). Zika Virus Replication in Myeloid Cells during Acute Infection Is Vital to Viral Dissemination and Pathogenesis in a Mouse Model. J. Virol..

[111] Wen C., Yu Y., Gao C., Qi X., Cardona C.J., Xing Z. (2022). Concomitant pyroptotic and apoptotic cell death triggered in macrophages infected by Zika virus. PLoS ONE.

[112] Antoniou E., Orovou E., Sarella A., Iliadou M., Rigas N., Palaska E., Iatrakis G., Dagla M. (2020). Zika Virus and the Risk of Developing Microcephaly in Infants: A Systematic Review. Int. J. Environ. Res. Public Health.

[113] King E.L., Irigoyen N. (2021). Zika Virus and Neuropathogenesis: The Unanswered Question of Which Strain Is More Prone to Causing Microcephaly and Other Neurological Defects. Front. Cell. Neurosci..

[114] Yang S., Gorshkov K., Lee E.M., Xu M., Cheng Y.S., Sun N., Soheilian F., de Val N., Ming G., Song H. (2020). Zika Virus-Induced Neuronal Apoptosis via Increased Mitochondrial Fragmentation. Front. Microbiol..

[115] Kuadkitkan A., Wikan N., Sornjai W., Smith D.R. (2020). Zika virus and microcephaly in Southeast Asia: A cause for concern?. J. Infect. Public Health.

[116] Gabriel E., Ramani A., Karow U., Gottardo M., Natarajan K., Gooi L.M., Goranci-Buzhala G., Krut O., Peters F., Nikolic M. (2017). Recent Zika Virus Isolates Induce Premature Differentiation of Neural Progenitors in Human Brain Organoids. Cell Stem Cell.

[117] Rosa-Fernandes L., Cugola F.R., Russo F.B., Kawahara R., de Melo Freire C.C., Leite P.E.C., Bassi Stern A.C., Angeli C.B., de Oliveira D.B.L., Melo S.R. (2019). Zika Virus Impairs Neurogenesis and Synaptogenesis Pathways in Human Neural Stem Cells and Neurons. Front. Cell. Neurosci..

[118] Li C., Xu D., Ye Q., Hong S., Jiang Y., Liu X., Zhang N., Shi L., Qin C.F., Xu Z. (2016). Zika Virus Disrupts Neural Progenitor Development and Leads to Microcephaly in Mice. Cell Stem Cell.

[119] Komarasamy T.V., Adnan N.A.A., James W., Balasubramaniam V. (2022). Zika Virus Neuropathogenesis: The Different Brain Cells, Host Factors and Mechanisms Involved. Front. Immunol..

[120] Williams A.L., Bohnsack B.L. (2015). Neural crest derivatives in ocular development: Discerning the eye of the storm. Birth Defects Res. C Embryo Today.

[121] Akula M., Park J.W., West-Mays J.A. (2019). Relationship between neural crest cell specification and rare ocular diseases. J. Neurosci. Res..

[122] Tamm E.R. (2011). Development of the iridocorneal angle and congenital glaucoma. Ophthalmologe.

[123] Tawara A., Inomata H. (1984). Developmental immaturity of the trabecular meshwork in juvenile glaucoma. Am. J. Ophthalmol..

[124] Alkatan H.M., Bedaiwi K.M., Al-Faky Y.H., Maktabi A.M.Y. (2022). Demographics and histopathological characteristics of enucleated microphthalmic globes. Sci. Rep..

[125] Verma A.S., Fitzpatrick D.R. (2007). Anophthalmia and microphthalmia. Orphanet J. Rare Dis..

[126] Williams A.L., Bohnsack B.L. (2020). The Ocular Neural Crest: Specification, Migration, and Then What?. Front. Cell Dev. Biol..

[127] Medina-Martinez O., Brownell I., Amaya-Manzanares F., Hu Q., Behringer R.R., Jamrich M. (2005). Severe defects in proliferation and differentiation of lens cells in Foxe3 null mice. Mol. Cell. Biol..

[128] El Costa H., Gouilly J., Mansuy J.M., Chen Q., Levy C., Cartron G., Veas F., Al-Daccak R., Izopet J., Jabrane-Ferrat N. (2016). ZIKA virus reveals broad tissue and cell tropism during the first trimester of pregnancy. Sci. Rep..

[129] Hirsch A.J., Roberts V.H.J., Grigsby P.L., Haese N., Schabel M.C., Wang X., Lo J.O., Liu Z., Kroenke C.D., Smith J.L. (2018). Zika virus infection in pregnant rhesus macaques causes placental dysfunction and immunopathology. Nat. Commun..

[130] Rabelo K., de Souza L.J., Salomao N.G., Machado L.N., Pereira P.G., Portari E.A., Basilio-de-Oliveira R., Dos Santos F.B., Neves L.D., Morgade L.F. (2020). Zika Induces Human Placental Damage and Inflammation. Front. Immunol..

[131] Singh P.K., Guest J.M., Kanwar M., Boss J., Gao N., Juzych M.S., Abrams G.W., Yu F.S., Kumar A. (2017). Zika virus infects cells lining the blood-retinal barrier and causes chorioretinal atrophy in mouse eyes. JCI Insight.

[132] Singh P.K., Khatri I., Jha A., Pretto C.D., Spindler K.R., Arumugaswami V., Giri S., Kumar A., Bhasin M.K. (2018). Determination of system level alterations in host transcriptome due to Zika virus (ZIKV) Infection in retinal pigment epithelium. Sci. Rep..

[133] Singh P.K., Singh S., Farr D., Kumar A. (2019). Interferon-stimulated gene 15 (ISG15) restricts Zika virus replication in primary human corneal epithelial cells. Ocul. Surf..

[134] Singh P.K., Kasetti R.B., Zode G.S., Goyal A., Juzych M.S., Kumar A. (2019). Zika Virus Infects Trabecular Meshwork and Causes Trabeculitis and Glaucomatous Pathology in Mouse Eyes. mSphere.

[135] Ueta M., Kawai T., Yokoi N., Akira S., Kinoshita S. (2011). Contribution of IPS-1 to polyI:C-induced cytokine production in conjunctival epithelial cells. Biochem. Biophys. Res. Commun..

[136] Mai K., Chui J.J., Di Girolamo N., McCluskey P.J., Wakefield D. (2014). Role of toll-like receptors in human iris pigment epithelial cells and their response to pathogen-associated molecular patterns. J. Inflamm..

[137] Ryan F.J., Carr J.M., Furtado J.M., Ma Y., Ashander L.M., Simoes M., Oliver G.F., Granado G.B., Dawson A.C., Michael M.Z. (2021). Zika Virus Infection of Human Iris Pigment Epithelial Cells. Front. Immunol..

[138] De Groot-Mijnes J.D.F., Chan A.S.Y., Chee S.P., Verjans G. (2018). Immunopathology of Virus-Induced Anterior Uveitis. Ocul. Immunol. Inflamm..

[139] Kalogeropoulos D., Sung V.C. (2018). Pathogenesis of Uveitic Glaucoma. J. Curr. Glaucoma Pract..

[140] Ventura C.V., Ventura L.O. (2018). Ophthalmologic Manifestations Associated With Zika Virus Infection. Pediatrics.

[141] Sherman K.E., Rouster S.D., Kong L.X., Aliota M.T., Blackard J.T., Dean G.E. (2019). Zika virus replication and cytopathic effects in liver cells. PLoS ONE.

[142] Kuo Y.P., Tsai K.N., Luo Y.C., Chung P.J., Su Y.W., Teng Y., Wu M.S., Lin Y.F., Lai C.Y., Chuang T.H. (2018). Establishment of a mouse model for the complete mosquito-mediated transmission cycle of Zika virus. PLoS Negl. Trop. Dis..

[143] Manangeeswaran M., Kielczewski J.L., Sen H.N., Xu B.C., Ireland D.D.C., McWilliams I.L., Chan C.C., Caspi R.R., Verthelyi D. (2018). ZIKA virus infection causes persistent chorioretinal lesions. Emerg. Microbes Infect..

[144] Stamer W.D., Clark A.F. (2017). The many faces of the trabecular meshwork cell. Exp. Eye Res..

[145] McArthur M.A. (2017). Zika Virus: Recent Advances towards the Development of Vaccines and Therapeutics. Viruses.

[146] Hoffman M., Chigbu D.I., Crumley B.L., Sharma R., Pustylnikov S., Crilley T., Ginwala R., Loonawat R., Joseph J., Sales D. (2020). Human Acute and Chronic Viruses: Host-Pathogen Interactions and Therapeutics. Advanced Concepts in Human Immunology: Prospects for Disease Control.

[147] Bodh S.A., Kumar V., Raina U.K., Ghosh B., Thakar M. (2011). Inflammatory glaucoma. Oman J. Ophthalmol..

[148] Badawi A.H., Al-Muhaylib A.A., Al Owaifeer A.M., Al-Essa R.S., Al-Shahwan S.A. (2019). Primary congenital glaucoma: An updated review. Saudi J. Ophthalmol..

[149] Tavallali A., Yannuzzi L.A. (2016). Idiopathic Multifocal Choroiditis. J. Ophthalmic Vis. Res..

[150] Baz M., Boivin G. (2019). Antiviral Agents in Development for Zika Virus Infections. Pharmaceuticals.

